
JOURNAL INFORMATION
==============================
NLM Title Abbreviation: Behav Neurol
Journal ID: Behav Neurol
NLM Journal ID: 8914585
Journal ID: BN

Behavioural Neurology

ISSN: 0953-4180
EISSN: 1875-8584
Publisher: Wiley

ARTICLE INFORMATION
==============================
PMCID: PMC5215062
PMID: 22713424
DOI: 10.3233/BEN-2012-129009
Article ID: 451346
Article version: 1
Subjects: Other

The Cognitive Safety of Deep Brain Stimulation in Refractory Psychiatric Disorders

Duits A. 1 3 *
Temel Y. 2 3
Ackermans L. 2 3
Visser-Vandewalle V. 2 3
1 Department of Psychiatry and Psychology Maastricht University Medical Centre Maastricht The Netherlands
2 Department of Neurosurgery Maastricht University Medical Centre Maastricht The Netherlands
3 School of Mental Health and NeuroScience Maastricht University Maastricht The Netherlands
*A. Duits: aa.duits@mumc.nl
Print publication date: 2013
Volume: 26
Issue: 3
First page: 195
Last page: 197
Received 2012 May 21; Accepted 2012 May 21
Copyright: Copyright © 2013 Hindawi Publishing Corporation and the authors.
Copyright year: 2013
License: This is an open access article distributed under the Creative Commons Attribution License, which permits unrestricted use, distribution, and reproduction in any medium, provided the original work is properly cited.
License URL: https://creativecommons.org/licenses/by/3.0/

==============================
Behavioural Neurology 26 (2013) 195–197
DOI 10.3233/BEN-2012-129009
IOS Press

### The cognitive safety of deep brain stimulation in refractory psychiatric disorders

A. Duits[a,c,*], Y. Temel[b,c], L. Ackermans[b,c] and V. Visser-Vandewalle[b,c]
[a]*Department of Psychiatry and Psychology, Maastricht University Medical Centre, Maastricht, The Netherlands*
[b]*Department of Neurosurgery, Maastricht University Medical Centre, Maastricht, The Netherlands*
[c]*School of Mental Health and NeuroScience, Maastricht University, Maastricht, The Netherlands*

Over the last decade, deep brain stimulation (DBS) has revolutionized the treatment of movement disorders and is now being used experimentally to treat refractory psychiatric disorders as well. DBS is a neurosurgical treatment involving the implantation of electrodes, which send electricity to specific targets deep in the brain. DBS in psychiatric disorders such as Tourette syndrome (TS), obsessive-compulsive disorder (OCD), major depression (MD) is based on focal neuromodulation of cortico-basal ganglia-thalamocortical loops involved in the control of behaviour and emotions paralleling the effect of modulating the motor circuit to treat movement disorders such as Parkinson's Disease (PD). DBS has crucial advantages over ablative procedures, performed mainly in the past, since it is reversible and adjustable [1]. However, it is not without risks and its cognitive safety is questionable. This study reviews the cognitive outcome of DBS in refractory TS, OCD and MD.

We searched PubMed and Medline for articles in English published up to December 2011 using combinations of the following terms: neuropsychological, cognitive, executive function, memory, attention, deep brain stimulation, neurosurgery, stereotaxy, TS, tics, OCD, MD, anxiety and mood. The retrieved abstracts were reviewed and the reference sections of the selected articles were screened for relevant studies.

*Corresponding author: Dr. AA Duits, Department of Psychiatry and Psychology, Maastricht University Medical Centre, PO Box 5800, 6202 AZ Maastricht, The Netherlands. Tel.: +31 43 3875686; Fax: +31 43 3875682; E-mail: aa.duits@mumc.nl.

Overall, the studies on DBS in TS, OCD and MD are based on small sample sizes and case reports, including follow-up data or (multicentre) combinations of original samples. Except for MD, relatively few studies reported on cognitive outcome. See Table 1 for the relevant studies. Owing to space considerations, case reports were excluded for review.

### 1. DBS in TS

TS is a neurodevelopmental disorder consisting of multiple motor and one or more vocal tics with onset in childhood. Comorbid symptoms include Attention Deficit Hyperactivity Disorder, OCD and obsessive-compulsive behaviour, self-injurious behaviour, depression and anxiety. These associated comorbidities are present in about 90% of clinically referred TS patients and have a significant impact on quality of life [12]. In a small number of patients, available treatment options such as pharmacological and cognitive behavioural therapy (CBT) are ineffective or cause intolerable side effects. When adults with severe tics are considered refractory to standard treatment, DBS may be an option. Since 1999, 25 studies including a total of 69 patients and 10 different targets have been published. In the majority of patients DBS resulted in significant tic reduction (50–99%) and in some cases there was also a positive effect on comorbidities [12]. Fatigue or reduced energy was the most reported DBS-related adverse effect [2,12]. Neuropsychologically (see Table 1), no changes have been found between baseline and as-

ISSN 0953-4180/13/$27.50 © 2013 – IOS Press and the authors. All rights reserved

Table 1
DBS and cognitive outcome in patients with refractory psychiatric disorders

| Syndrome | Target | Design | Cognitive outcome | Reference |
|---|---|---|---|---|
| TS | Thalamus | double-blind/open-label, $n = 6$, 12 months FU | decrease in selective attention | [2] |
| TS | Thalamus | open-label, $n = 15$, 24 months FU | no decline but improvement in mental speed/ cognitive flexibility | [3] |
| TS | Thalamus/GPi | controlled double-blind randomized crossover $n = 3$, up to 10 months | no changes in all (4) conditions | [4] |
| OCD | VC/VS | randomized staggered onset, double-blind, $n = 6$, 6 and 12 months FU | no changes at 6 and 12 months | [5] |
| OCD | VC/VS | open-label, $n = 10$, FU cognition at 10 months | no individual changes but improvement of group in recall of prose passages | [6] |
| OCD | ALIC | double-blind/open-label, $n = 4$, 6 months FU | no changes at a group level | [7] |
| MD | SCG | open-label, $n = 8$ FU cognition at 6 months | no change in test scores | [8] |
| MD | NAc | open-label, $n = 10$ 12 months FU | improvement at a group level in attention, learning and memory, executive function, visual perception | [9] |
| MD | VC/VS | open-label, $n = 10$ FU cognition at 6 months | improvement at a group level in verbal memory (logical memory delay) | [10] |
| MD | SCG | open-label, $n = 6$ FU cognition at 12 months | no change based on reliable change indices | [11] |

TS = Tourette syndrome; FU = follow-up; GPi = globus pallidus internus; OCD = obsessive compulsive disorder; VC/VS = ventral capsule/ventral striatum; ALIC = anterior limb of the internal capsule; MD = major depression; SCG = subcallosal cingulate gyrus; NAc = nucleus accumbens.

sessment up to two years after surgery, except for an improvement in mental speed/cognitive flexibility [3] and a decline in selective attention and response inhibition [2]. However, reliable change indices showed a decline in only one of the six patients [2].

### 2. DBS in OCD

OCD is a heterogeneous, chronic, and disabling anxiety disorder, characterized by disturbing intrusive thoughts (obsessions) and repetitive ritualized behaviours (compulsions) aimed at reducing anxiety and discomfort. OCD significantly interferes with an individual's daily routine and has considerable percussions on family and social life and occupational functioning. It is estimated that about 10% of patients remains severely affected despite optimal pharmacological therapy and CBT. In total about 100 refractory OCD patients have been reported with DBS in five different targets. Overall, the reported success rates were $> 50\%$. Chronic mood improvement appeared to be a favourable side effect of DBS, whereas transient hypomania was the most reported adverse effect [13]. DBS has not been associated with evident cognitive decline or improvement (see Table 1) but subjective data with complaints of forgetfulness and word finding have been reported following bilateral stimulation of the nucleus accumbens (NAc) [14].

### 3. DBS in MD

MD is a complex, multisystem illness characterized by persistently depressed mood or decreased interest in usual activities accompanied by difficulty concentrating, insomnia or hypersomnia, psychomotor retardation or agitation, weight gain or loss, increased or decreased appetite, or feelings of worthlessness or guilt. Even though many patients with depression will respond well to pharmacological treatment, psychotherapy and electroconvulsive therapy, a significant proportion of patients is refractory to treatment. So far, studies with DBS in five different targets have been published, especially in the subcallosal cingulate gyrus (SCG). About 50% of patients responded with a substantial reduction (40–50%) in depressive symptoms during acute and long-term bilateral stimulation but some cases with increased levels of depression, hypomania and suicide (attempts) have also been reported [15]. In general, patients suffering from depression frequently show cognitive disturbances, which emphasizes the importance of treatments with cognitive safety. So far, studies of DBS in MD did not reveal any evidence for DBS-associated cognitive decline (see Table 1). Indeed, significant cognitive improvement occurred independently of a reduction of depressive symptoms and changes in stimulation parameters. Especially the NAc might have an independent augmenting effect on cognition [9], but cognitive improvement was also found in patients with

SCG-DBS [11]. However, this improvement was not significant in terms of reliable change indices.

In conclusion, the results of DBS in TS, OCD and MD show different but substantial degrees of success and side effects were mostly transient, relatively well tolerated or inverted by changing stimulus parameters [1]. Relatively few studies, especially in case of TS and OCD, reported on cognitive outcome and all sample sizes were relatively small with variation in neuropsychological tests used. Overall, DBS has not been associated with clinically significant cognitive decline, providing support for its cognitive safety. Moreover, improved cognitive performance, independent of the antidepressant effect, was found in patients with DBS for MD, especially when stimulating the NAc.

Given the small number of studies and their limited power, the cognitive consequences of DBS in refractory psychiatric disorders need to be further studied. Like in DBS for PD [1], reduced impulse control underlies the serious adverse events, which may have implications for cognition.

### References

[1] P. Krack, M.I. Hariz, C. Baunez, J. Guridi and J.A. Obeso, Deep brain stimulation: from neurology to psychiatry? *Trends Neurosci* **33** (2010), 474–484.

[2] L. Ackermans, A. Duits, C. Van der Linden, M. Tijssen, K. Schruers, Y. Temel et al., Double-blind clinical trial of thalamic stimulation in six patients with Tourette Syndrome, *Brain* **134** (2011), 832–844.

[3] M. Porta, A. Brambilla, A.E. Cavanna, D. Servello, M. Sassi, H. Rickards et al., Thalamic deep brain stimulation for treatment-refractory Tourette Syndrome: two-year outcome, *Neurology* **73** (2009), 1375–1380.

[4] M. Welter, L. Mallet, J. Houeto, C. Karachi, V. Czernecki, P. Cornu et al., Internal pallidal and thalamic stimulation in patients with Tourette syndrome, *Arch Neurol* **65** (2008), 952–957.

[5] W.K. Goodman, K.D. Foote, B.D. Greenberg, N. Ricciuti, R. Bauer, H. Ward et al., Deep brain stimulation for intractable obsessive compulsive disorder: pilot study using a blinded, staggered-onset design, *Biol Psychiatry* **67** (2010), 535–542.

[6] B.D. Greenberg, D.A. Malone, G.M. Friehs, A.R. Rezai, C.S. Kubu, P.F. Malloy et al., Three-year outcomes in deep brain stimulation for highly resistant obsessive-compulsive disorder, *Neuropsychopharmacology* **31**(11) (2006), 2384–2393.

[7] J.L. Abelson, G.C. Curtis, O. Sagher, R.C. Albucher, M. Harrigan, S.F. Taylor et al., Deep brain stimulation for refractory obsessive compulsive disorder, *Biol Psychiatry* **57**(5) (2005), 510–516.

[8] D. Puigdemont, R. Pérez-Egea, M.J. Portella, J. Molet, J. de Diego-Adeliño, A. Gironell et al., Deep brain stimulation of the subcallosal cingulate gyrus: further evidence in treatment-resistant major depression, *Int J Neuropsychopharmacol* **22** (2011), 1–13.

[9] C. Grubert, R. Hurlemann, B.H. Bewernick, S. Kayser, B. Hadrysiewicz, N. Axmacher et al., Neuropsychological safety of nucleus accumbens deep brain stimulation for major depression: effects of 12-month stimulation, *World J Biol Psychiatry* **12**(7) (2011), 516–527.

[10] D.A. Malone, D.D. Dougherty, A.R. Rezai, L.L. Carpenter, G.M. Friehs, E.N. Eskandar et al., Deep brain stimulation of the ventral capsule/ventral striatum for treatment-resistant depression, *Biol Psychiatry* **65** (2009), 267–275.

[11] H.E. McNeely, H.S. Mayberg, A.M. Lozano and S.H. Kennedy, Neuropsychological impact of Cg25 deep brain stimulation for treatment-resistant depression: preliminary results over 12 months, *J Nerv Ment Dis* **196** (2008), 405–410.

[12] K.R. Muller-Vahl, D.C. Cath, A.E. Cavanna, S. Dehning, M. Porta, M.M. Robertson et al., the ESSTS Guidelines Group. European clinical guidelines for Tourette syndrome and other tic disorders. Part IV: deep brain stimulation, *Eur Child Adolesc Psychiatry* **20** (2011), 209–217.

[13] P.P. de Koning, M. Figee, P. van den Munckhof, P.R. Schuurman and D. Denys, Current status of deep brain stimulation for obsessive-compulsive disorder: a clinical review of different targets, *Curr Psychiatry Rep* **13** (2011), 274–282.

[14] D. Denys, M. Mantione, M. Figee, P. van den Munckhof, F. Koerselman, H. Westenberg et al., Deep brain stimulation of the nucleus accumbens for treatment-refractory obsessive-compulsive disorder, *Arch Gen Psychiatry* **67**(10) (2010), 1061–1068.

[15] W.K. Goodman and R.L. Alterman, Deep brain stimulation for intractable psychiatric disorders, *Annu Rev Med* **63** (2012), 511–524.
